# *HLA-DRB3/4/5* Matching Improves Outcome of Unrelated Hematopoietic Stem Cell Transplantation

**DOI:** 10.3389/fimmu.2021.771449

**Published:** 2021-12-14

**Authors:** Chrysanthi Tsamadou, Daphne Engelhardt, Uwe Platzbecker, Elisa Sala, Thomas Valerius, Eva Wagner-Drouet, Gerald Wulf, Nicolaus Kröger, Niels Murawski, Hermann Einsele, Kerstin Schaefer-Eckart, Sebastian Freitag, Jochen Casper, Martin Kaufmann, Mareike Dürholt, Bernd Hertenstein, Stefan Klein, Mark Ringhoffer, Sandra Frank, Christine Neuchel, Hubert Schrezenmeier, Joannis Mytilineos, Daniel Fuerst

**Affiliations:** ^1^ Institute of Clinical Transfusion Medicine and Immunogenetics Ulm, German Red Cross Blood Transfusion Service, Baden Wuerttemberg – Hessen, and University Hospital Ulm, Ulm, Germany; ^2^ Institute of Transfusion Medicine, University of Ulm, Ulm, Germany; ^3^ Department of Otorhinolaryngology, Head and Neck Surgery, University of Ulm, Ulm, Germany; ^4^ Department of Hematology/Oncology, University of Leipzig, Leipzig, Germany; ^5^ Department of Internal Medicine III, University of Ulm, Ulm, Germany; ^6^ Section for Stem Cell Transplantation and Immunotherapy, Department of Medicine II, Christian Albrechts University, Kiel, Germany; ^7^ Department of Medicine III, Johannes Gutenberg-University, Mainz, Germany; ^8^ Department of Hematology/Oncology, Georg-August-University, Göttingen, Germany; ^9^ Department of Stem Cell Transplantation, University Hospital Hamburg Eppendorf, Hamburg, Germany; ^10^ Department of Internal Medicine I, Universitätsklinikum des Saarlandes, Homburg, Germany; ^11^ Department of Internal Medicine II, University Hospital Würzburg, Würzburg, Germany; ^12^ Medical Clinic 5: Hematology, Oncology, Nuremberg Hospital, Nuremberg, Germany; ^13^ Department of Medicine III, Hematology/Oncology/Palliative Care, Rostock University Medical Center, Rostock, Germany; ^14^ Department of Oncology and Hematology, Klinikum Oldenburg, University Clinic, Oldenburg, Germany; ^15^ 2nd Department of Internal Medicine, Oncology and Hematology, Robert Bosch Hospital, Stuttgart, Germany; ^16^ Hematology/Oncology, Evangelic Clinic Essen-Werden, Essen-Werden, Germany; ^17^ Hematology/Oncology, Klinikum Bremen-Mitte, Bremen, Germany; ^18^ Universitätsmedizin Mannheim, Med. Klinik III, Mannheim, Germany; ^19^ Medizinische Klinik III, Städtisches Klinikum Karlsruhe, Karlsruhe, Germany; ^20^ DRST –Deutsches Register für Stammzelltransplantationen, German Registry for Stem Cell Transplantation, Ulm, Germany; ^21^ ZKRD–Zentrales Knochenmarkspender-Register für Deutschland, German National Bone Marrow Donor Registry, Ulm, Germany

**Keywords:** *HLA-DRB3*, *HLA-DRB4*, *HLA-DRB5*, *HLA-DRB3/4/5*, unrelated hematopoietic stem cell transplantation (uHSCT), HLA-matched, DRB3/4/5 matching

## Abstract

The *HLA-DRB3/4/5* loci are closely linked to the *HLA-DRB1* gene. Mismatches in these loci occur with a frequency of about 8%–12% in otherwise 10/10 HLA-matched transplant pairs. There is preliminary evidence that these disparities may associate with increased acute graft-versus-host disease (GvHD) rates. The aim of this study was to analyze a large cohort of German patients and their donors for *HLA-DRB3/4/5* compatibility and to correlate the *HLA-DRB3/4/5* matching status with the outcome of unrelated hematopoietic stem cell transplantation (uHSCT). To this end, 3,410 patients and their respective donors were *HLA-DRB3/4/5* and *HLA-DPB1* typed by amplicon-based next-generation sequencing (NGS). All patients included received their first allogeneic transplant for malignant hematologic diseases between 2000 and 2014. Mismatches in the antigen recognition domain (ARD) of *HLA-DRB3/4/5* genes were correlated with clinical outcome. *HLA-DRB3/4/5* incompatibility was seen in 12.5% (n = 296) and 17.8% (n = 185) of the 10/10 and 9/10 HLA-matched cases, respectively. *HLA-DRB3/4/5* mismatches in the ARD associated with a worse overall survival (OS), as shown in univariate (5-year OS: 46.1% vs. 39.8%, log-rank p = 0.038) and multivariate analyses [hazard ratio (HR) 1.25, 95% CI 1.02–1.54, p = 0.034] in the otherwise 10/10 HLA-matched subgroup. The worse outcome was mainly driven by a significantly higher non-relapse mortality (HR 1.35, 95% CI 1.05–1.73, p = 0.017). In the 9/10 HLA-matched cases, the effect was not statistically significant. Our study results suggest that mismatches within the ARD of *HLA-DRB3/4/5* genes significantly impact the outcome of otherwise fully matched uHSCT and support their consideration upon donor selection in the future.

## Introduction

*HLA-DRB3/4/5* genes are closely linked to the *HLA-DRB1* locus (1). They exhibit a strong linkage disequilibrium in often conserved HLA-class II haplotypes (2). Within such haplotypes, the *HLA-DRB1* antigen determines the presence or absence of an *HLA-DRB3/4/5* gene. The three loci *HLA-DRB3*, *HLA-DRB4*, and *HLA-DRB5* show a lower expression as compared to *HLA-DRB1* but are still detectable by serological methods on account of which they are designated as *HLA-DR52*, -DR53, and -DR51 antigens, respectively (3). The *HLA-DR51* (i.e., DRB5*) antigen is commonly present when HLA-DRB1*15 or -DRB1*16 alleles are also present. The *HLA-DR52* (i.e., DRB3*) antigen is linked to HLA-DRB1*03, *11, *12, *13, and *14 alleles, while the *HLA-DR53* (i.e., DRB4*) antigen is found together with HLA-DRB1*04, *07, and *09. Generally, alleles from the HLA-DRB1*01, *08, and *10 antigen groups do not associate with any of the *HLA-DRB3/4/5* genes (4). Additionally, non-expressed alleles are quite frequent within the *HLA-DRB3/4/5* genes, particularly HLA-DRB4*01:03:01:02N, which is the most frequent HLA-null allele recognized with an overall allele frequency of about 3.5% (5, 6). Despite the strong linkage disequilibrium, unusual *HLA-DRB1-DRB3/4/5* associations do occur, leading to unexpected absence or presence of an *HLA-DR52/53/51* antigen. This is sometimes observed in HLA-DRB1*01 that occasionally associates with HLA-DRB5 (7). In addition, *HLA-DRB3/4/5* typing can be challenging due to the presence of unusual associations and non-expressed variants caused by polymorphisms in non-coding regions (e.g., HLA-DRB4*01:03:01:02N or HLA-DRB4*01:14N) (8, 9).

Due to the strong linkage between *HLA-DRB1* and *HLA-DRB3/4/5*, mismatches at the *HLA-DRB3/4/5* loci have been reported in only a fraction of transplant pairs (10, 11) with frequencies between 9.5% and 12.8% in different retrospective cohorts (10–12). The relevance of *HLA-DRB3/4/5* discrepancies on outcome of uHSCT is still controversial. Some case studies showed that severe graft-versus-host disease (GvHD) may be induced by mismatches in these genes, while others speculated that the lower level of expression precludes any significant effects (12, 13). That said, preliminary cohort studies suggest that *HLA-DRB3/4/5 differences do associate with increased mortality and GvHD* events. There is evidence that factors beyond expression may influence alloreactivity such as, for instance, the cumulative number of mismatches and/or the direction of mismatches (i.e., antigen missing in donor or recipient or bidirectional if different allotypes in patient and donor are present (12, 14).

In order to investigate the potential role of *HLA-DRB3/4/5* differences in GvHD incidence and mortality after uHSCT, we retrospectively analyzed a large cohort of German transplant pairs for differences within the antigen recognition domain (ARD) (exon 2) and/or exon 3 of HLA-DRB4 genes and correlated these mismatches with outcome.

## Patients and Methods

### Study Population

This study included a total of 3,410 patients who underwent first allo-HSCT between 2000 and 2014 for malignant hematological diseases (i.e., acute and chronic leukemia, Myelodysplastic syndrome (MDS), Non-Hodgkin Lymphoma (NHL), and myeloma) with peripheral blood stem cells (PBSCs) or bone marrow (BM) from an unrelated donor at German transplant centers. Stem cell donor searches for cooperating transplant centers were conducted by the search unit in Ulm.

### Clinical Data

Clinical data were obtained from the German registry for stem cell transplantation (DRST) that is a subset of the EBMT ProMISe database for German patients. Treatment decisions and follow-up information from day 0, day 100, and yearly afterward were collected by the cooperating transplant centers based on EBMT surveys (MED-AB-Survey). Missing data in the EBMT files were retrieved directly from the centers when possible.

### HLA Typing

In the stem cell donor search setting, high-resolution HLA typing of the classical HLA gene loci HLA-A, -B, -C, -DRB-1, and -DQB1 had already been performed up front in the tissue typing laboratory of the search unit in Ulm. Retrospective typing for HLA-DPB1 and *HLA-DRB3/4/5* loci was performed using an in-house-developed CE-certified next-generation sequencing (NGS)-amplicon sequencing protocol based on the Illumina MiSeq platform (San Diego, CA, USA) (15) in both patients and donors (n = 6,820). Matching status between donor and patient was defined by the identity of the protein sequence in the ARD, which is determined by exon 2 sequences for these loci (16). Additionally, differences in HLA-DRB4 exon 3 were determined and evaluated separately in this analysis.

### Definitions

Definition of the disease status prior to transplantation was classified according to definitions previously used by the EBMT study group (17). Myeloablative conditioning (MAC) was classified according to the EBMT MED-AB manual Appendix III and published consensus suggestions and compared to less intense regimen termed reduced-intensity conditioning (RIC) (18). The primary endpoints for the analysis in this study were defined according to the EBMT statistical recommendations (19). Overall survival (OS) was defined as the time from stem cell transplantation to death or last follow-up. Disease-free survival (DFS) was defined as the time to relapse or death from any cause or last follow-up. GvHD and relapse-free survival (GRFS) was defined as the time from the transplantation to the development of acute GvHD (aGvHD), relapse, or death, whichever occurred first. Non-relapse mortality (NRM) was defined as the time from transplantation until any cause of death without previous relapse, with disease relapse serving as a competing risk. Acute GvHD incidence was defined as the time to first occurrence of aGvHD grades II–IV, with death from other causes and disease relapse constituting competing risks. Relapse incidence was defined as the time to the event of a relapse, with death from other causes as a competing risk.

### Statistics

Statistical analysis of patient characteristics was performed by chi-square test for categorical variables and Mann–Whitney U test for continuous variables. For univariate analysis, Kaplan–Meier analysis with log-rank testing was used. Cox proportional hazards regression models were used for multivariate analysis of the endpoints OS, DFS, and GRFS. For the endpoints NRM, aGvHD, chronic GvHD (cGvHD), and relapse, competing risk regression according to the Fine and Gray model was used (20). Models were stratified for diagnosis and included adjustments for a center effect. Models for the variables of interests were checked for interactions, and none was found. Significance level was set at p = 0.05.

### Study Design and Ethics

This study is a retrospective analysis of the impact of *HLA-DRB3/4/5* matching status between patient and donor on the outcome after uHSCT. The endpoints mentioned above were analyzed in the context of relevant clinical and immunobiological variables. All patients and donors provided consent for HLA typing. Consent for recording and scientific analysis of the clinical data was obtained prior to registration of patients in the EBMT ProMISe database. The study was approved by the ethical committee of the University of Ulm.

## Results

Patients’ characteristics with respect to overall HLA and HLA-DRB3/4/5 compatibility are summarized in **Table 1**. The distribution as to clinical predictors within the respective subgroups was balanced, with only mild differences observed for HLA-DPB1 matching status (p = 0.035 and 0.037 for the 10/10 and 9/10 HLA-matched cohorts, respectively). Median patient age for the whole cohort was 54 years, and acute myeloid leukemia (AML) was the most frequent diagnosis (n = 1,255, 36.8%). Disease status at the time of transplantation was for most of the patients either early stage (n = 1,343, 39.4%) or intermediate stage (n = 1,177, 34.5%). The majority of the patients received *in vivo* T-cell depletion consisting of anti-thymocyte globulin (ATG) or alemtuzumab (n = 2,273, 66.7%). MAC was performed in 62.2% of the patients (n = 2,122). In this cohort, 2,371 (69.5%) patients underwent a 10/10 HLA-matched transplantation, while 1,039 (30.5%) patients received a 9/10 HLA-matched transplant. Median follow-up time was 52 months.

**Table 1:** Cohort characteristics.

	10/10 DRB345 M n = 2075	10/10 DRB345 MM n = 296	p-value	9/10 DRB345 M n = 854	9/10 DRB345 MM n = 185	p-value
**Age**	54 (1–75)	53 (1–77)	0.152	53 (2–76)	54 (1–74)	0.161
**AML**	739 (35.6)	114 (38.5)	0.638	332 (38.9)	70 (37.8)	0.086
**MDS**	331 (16)	43 (14.5)		125 (14.6)	36 (19.5)	
**NHL**	269 (13)	44 (14.9)		102 (11.9)	11 (5.9)	
**ALL**	237 (11.4)	36 (12.2)		109 (12.8)	22 (11.9)	
**MM**	204 (9.8)	27 (9.1)		80 (9.4)	14 (7.6)	
**CLL**	122 (5.9)	13 (4.4)		42 (4.9)	12 (6.5)	
**AL**	110 (5.3)	10 (3.4)		32 (3.7)	13 (7)	
**CML**	63 (3)	9 (3)		32 (3.7)	7 (3.8)	
**Early-stage disease**	795 (38.3)	127 (42.9)	0.118	337 (39.5)	84 (45.4)	0.108
**Intermediate-stage disease**	728 (35.1)	106 (35.8)		294 (34.4)	49 (26.5)	
**Advanced-stage disease**	552 (26.6)	63 (21.3)		223 (26.1)	52 (28.1)	
**Donor age 18–30**	720 (34.7)	97 (32.8)	0.011	238 (27.9)	63 (34.1)	0.406
**Donor age 31–45**	1,000 (48.2)	137 (46.3)		392 (45.9)	76 (41.1)	
**Donor age 46–60**	310 (14.9)	46 (15.5)		167 (19.6)	34 (18.4)	
**Missing**	45 (2.2)	16 (5.4)		57 (6.7)	12 (6.5)	
**HLA-DPB1 matched**	457 (22)	54 (18.2)	0.035	162 (19)	31 (16.8)	0.037
**HLA-DPB1 permissive**	786 (37.9)	106 (35.8)		306 (35.8)	54 (29.2)	
**HLA-DPB1 non-permissive**	832 (40.1)	135 (45.6)		386 (45.2)	99 (53.5)	
**Missing**	0 (0)	1 (0.3)		0 (0)	1 (0.5)	
**Year of Tx 2000–2003**	24 (1.2)	4 (1.4)	0.294	26 (3)	1 (0.5)	0.022
**Year of Tx 2004–2009**	921 (44.4)	118 (39.9)		442 (51.8)	84 (45.4)	
**Year of Tx 2010–2014**	1,130 (54.5)	174 (58.8)		386 (45.2)	100 (54.1)	
***In vivo* T-cell depletion**	1,400 (67.5)	193 (65.2)	0.689	557 (65.2)	123 (66.5)	0.911
**No *in vivo* T-cell depletion**	445 (21.4)	66 (22.3)		157 (18.4)	34 (18.4)	
**Missing**	230 (11.1)	37 (12.5)		140 (16.4)	28 (15.1)	
**KPS 80–100**	1,611 (77.6)	217 (73.3)	0.251	590 (69.1)	132 (71.4)	0.601
**KPS <80**	91 (4.4)	16 (5.4)		47 (5.5)	12 (6.5)	
**Missing**	373 (18)	63 (21.3)		217 (25.4)	41 (22.2)	
**MAC**	1,268 (61.1)	176 (59.5)	0.251	565 (66.2)	113 (61.1)	0.601
**RIC**	806 (38.8)	120 (40.5)		289 (33.8)	72 (38.9)	
**Missing**	1 (0)	0 (0)		0 (0)	0 (0)	
**P-D CMV neg-neg**	664 (32)	96 (32.4)		235 (27.5)	54 (29.2)	
**P-D CMV neg-pos**	176 (8.5)	23 (7.8)		88 (10.3)	18 (9.7)	
**P-D CMV pos-neg**	473 (22.8)	76 (25.7)	0.565	247 (28.9)	54 (29.2)	0.861
**P-D CMV pos-pos**	653 (31.5)	91 (30.7)		235 (27.5)	52 (28.1)	
**Missing**	109 (5.3)	10 (3.4)		49 (5.7)	7 (3.8)	
**BM**	104 (5)	19 (6.4)		59 (6.9)	19 (10.3)	
**PBSC**	1,971 (95)	277 (93.6)	0.378	794 (93.1)	166 (89.7)	0.157

AL, acute leukemia undifferentiated, biphenotypic or secondary; M, match; MM, mismatch; KPS, Karnofsky Performance Score; MAC, myeloablative conditioning; RIC, reduced-intensity conditioning.

Due to the diversity of HLA-class II haplotypes, different mismatch combinations had to be considered in the analyses. In 10/10 matched transplantations, 2,075 (87.5%) were *HLA-DRB3/4/5* matched and 296 (12.5%) mismatched. A similar distribution was observed in the 9/10 group, with 854 (82.2%) *HLA-DRB3/4/5* matched and 185 (17.8%) *HLA-DRB3/4/5* mismatched transplantations. Identified *HLA-DRB3/4/5* mismatches occurred within the ARD of HLA-DRB3/4/5 and/or HLA-DRB4 exon 3. Current consensus is that mismatches in the ARD are clinically relevant, so only differences within the ARD were included in the univariate models. As mismatches in *HLA-DRB4* exon 3 were suggested as relevant in one previous study^12^, we incorporated *HLA-DRB4* exon 3 mismatches as a separate variable in multivariate analysis to assess any additional risk conferred by such differences. In an overall 481 identified *HLA-DRB3/4/5* mismatched cases, 334 (69.4%) concerned the ARD (exon 2) and 147 (30.6%) related to differences in HLA-DRB4 exon 3 only. Some ARD mismatches were combined with additional *HLA-DRB4* exon 3 mismatches (**Table 2**). Mismatches due to missing or additional antigens were classified as ARD discrepancies. These were most often caused by the presence of HLA-DRB4*01:03:01:02N in donor or recipient (n = 29, 0.85%, missing antigen) and due to the uncommon association of *HLA-DRB5* with HLA-DRB1*01 (n = 9, 0.27%, additional antigen). A detailed overview of the mismatch combinations is given in **Table 2**.

**Table 2:** HLA-DRB345 mismatch status.

DRB345 Status	HLA 10/10	HLA 9/10	Total
**3ARD**	174 (7.3)	90 (8.7)	264
**3ARD, 3ARD**	1 (0)	1 (0.1)	2
**3ARD, 4ARD**	0 (0)	1 (0.1)	1
**3ARD, 4E3**	8 (0.3)	4 (0.4)	12
**3ARD, 4E3, 5ARD**	0 (0)	1 (0.1)	1
**3ARD, 5ARD**	1 (0)	0 (0)	1
**4ARD**	10 (0.4)	30 (2.9)	40
**4ARD, 4ARD**	1 (0)	0 (0)	1
**4E3**	95 (4)	52 (5)	147
**5ARD**	6 (0.3)	6 (0.6)	12
**DRB345 match**	2,075 (87.5)	854 (82.2)	2,929
**Total**	2,371	1,039	3,410

The leading number denotes the gene DRB3, DRB4, or DRB5. ARD is a mismatch within the antigen recognition domain. E3 is a mismatch within exon 3. Concomitant mismatches are separated by commas. ARD, antigen recognition domain; E3, exon 3.

**Figure 1:**
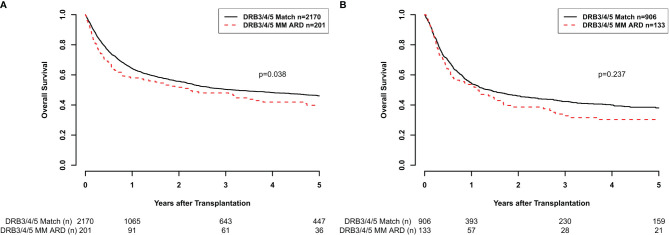
**(A)** Overall survival (OS) according to *HLA-DRB3/4/5* matching status (M = match, MM = mismatch) considering the antigen recognition domain (ARD) only in the subgroup of 10/10-matched transplant pairs (p = 0.038). **(B)** OS according to *HLA-DRB3/4/5* matching status (M, MM) considering the ARD only in the subgroup of 9/10-matched transplant pairs (p = 0.237).

OS in univariate analysis showed a significantly better outcome for 10/10 HLA-A, -B, -C, -DRB1, and -DQB1-matched transplants when *HLA-DRB3/4/5* were also matched compared to *HLA-DRB3/4/5* ARD-mismatched (MM) cases (log-rank p = 0.038; **Figure 1A**). Estimates at 1, 3, and 5 years posttransplantation were 64.2% (62.1–66.5), 50.4% (48.0–52.8), and 46.1% (43.6–48.6) for the *HLA-DRB3/4/5*-matched group vs. 58.0% (51.1–65.7), 48.0% (40.9–56.3), and 39.8% (32.5–48.7) for the *HLA-DRB3/4/5* ARD-MM group. This association was not statistically significant in the subgroup of 9/10 HLA-matched transplanted patients, n = 1,039 (log-rank p = 0.237; **Figure 1B**). Specifically, in this subgroup (n = 1,039), estimates for OS at 1, 3, and 5 years posttransplantation were at 54.5% (51.2–58.1), 42.3% (38.9–46.1), and 38.0% (34.5–41.9) for the HLA-DRB345-matched group vs. 52.7% (44.4–62.7), 32.8% (24.8–43.3), and 30.3% (22.5–40.9) for the *HLA-DRB3/4/5* ARD-MM group.

In the multivariate analysis of 10/10 HLA-matched cases, *HLA-DRB3/4/5* ARD-MM showed a significantly higher overall mortality risk [OS: hazard ratio (HR) 1.25, 95% CI 1.02–1.54, p = 0.034; **Table 3**]. For DFS and GFRS, no significant differences were seen between the groups (**Table 3**). Results for 9/10-matched cases and combined models concerning *HLA-DRB3/4/5* ARD and HLA-DRB4 E3 differences are shown in the **Supplementary Material**.

**Table 3:** Survival endpoints 10/10 HLA-matched group.

	OS		DFS		GRFS	
	HR (95% CI)	p-value	HR (95% CI)	p-value	HR (95% CI)	p-value
**Patient age**	1.01 (1.01–1.02)	**<0.001**	1.01 (1.01–1.02)	**<0.001**	1.01 (1.00–1.01)	**<0.001**
**Donor age 18–30**	1.00		–		–	
**Donor age 31–45**	1.18 (1.03–1.35)	**0.020**	**-**		**-**	
**Donor age 46–60**	1.22 (1.01–1.48)	**0.035**	**-**		**-**	
**Early stage disease**	1.00		1.00		1.00	
**Intermediate-stage disease**	1.20 (1.04–1.40)	**0.015**	1.32 (1.14–1.52)	**<0.001**	1.29 (1.13–1.47)	**<0.001**
**Advanced-stage disease**	1.84 (1.59–2.14)	**<0.001**	1.88 (1.63–2.16)	**<0.001**	1.56 (1.37–1.78)	**<0.001**
***HLA-DRB3/4/5* M, n = 2,075**	1.00		1.00		1.00	
***HLA-DRB3/4/5* ARD MM, n = 201**	1.25 (1.02–1.54)	**0.034**	1.17 (0.97–1.41)	0.110	1.13 (0.95–1.35)	0.160
**HLA-DRB4 E3 M, n = 2,268**	1.00		1.00		1.00	
**HLA-DRB4 E3 MM, n = 103**	1.04 (0.76–1.40)	0.822	0.98 (0.75–1.30)	0.912	1.16 (0.91–1.47)	0.230
**HLA-DPB1 Match/Permissive**	–		–		1.00	
**HLA-DPB1 Non-Permissive**	**-**		**-**		1.12 (1.01–1.23)	**0.031**
**Year of Tx 2000–2003**	1.00		–		1.00	
**Year of Tx 2004–2009**	0.67 (0.42–1.09)	0.105	–		0.55 (0.36–0.85)	**0.007**
**Year of Tx 2010–2014**	0.64 (0.39–1.05)	0.080	–		0.55 (0.35–0.85)	**0.008**
***In vivo* TCD**	1.00		1.00		1.00	
**No *in vivo* TCD**	1.24 (1.04–1.48)	**0.018**	1.19 (1.02–1.39)	**0.032**	1.21 (1.05–1.39)	**0.009**
**KPS 80–100**	1.00		1.00		1.00	
**KPS <80**	1.52 (1.14–2.02)	**0.005**	1.66 (1.29–2.14)	**<0.001**	1.36 (1.08–1.71)	**0.009**
**RIC**	1.00		1.00		–	
**MAC**	1.20 (1.04–1.40)	**0.014**	1.21 (1.06–1.39)	**0.005**	**-**	
**P-D CMV neg-neg**	1.00		1.00		1.00	
**P-D CMV neg-pos**	1.16 (0.92–1.47)	0.218	1.24 (1.01–1.51)	**0.040**	1.14 (0.95–1.38)	0.164
**P-D CMV pos-neg**	1.11 (0.93–1.31)	0.241	1.06 (0.91–1.23)	0.459	0.98 (0.85–1.12)	0.734
**P-D CMV pos-pos**	1.07 (0.92–1.25)	0.377	1.06 (0.93–1.22)	0.370	0.98 (0.86–1.11)	0.748

OS, overall survival; DFS, disease-free survival; GvHD, graft-versus-host disease; GRFS, GvHD and disease-free survival; M, match; MM, mismatch; ARD, antigen recognition domain; TCD, T-cell depletion; KPS, Karnofsky Performance Score; RIC, reduced-intensity conditioning; MAC, myeloablative conditioning; D, patient-donor; KIR C1x/C2C2, KIR ligand group.

Statistical significance is marked in bold.

The aforementioned adverse survival outcomes were predominantly caused by a higher risk of NRM in *HLA-DRB3/4/5* ARD-mismatched cases (NRM: 1.35, 95% CI 1.05–1.73, p = 0.017; **Table 4**). No statistically significant risk estimates were observed regarding the incidence rates of aGvHD and cGvHD (aGvHD II–IV: HR 1.16, 95% CI 0.89–1.52, p = 0.277 and cGvHD: HR 1.12, 95% CI 0.85–1.47, p = 0.433). Additional analysis focused on severe aGvHD (i.e., III–IV) incidence rates also failed to reveal any significant association (aGvHD III–IV: HR 1.19, 95% CI 0.79–1.79, p = 0.412). Similarly, exon 3 mismatches at HLA-DRB4 were not associated with a significantly increased risk (**Table 4**). However, grouping ARD and exon 3 mismatches together resulted in a significantly higher aGvHD risk in a model encompassing all transplant pairs (aGvHD II–IV: HR 1.21, 95% CI 1.02–1.43, p = 0.027; **Supplementary Material**). No significant difference on relapse rates could be identified for *HLA-DRB3/4/5* mismatches, regardless of gene region concerned (i.e., ARD or HLA-DRB4 exon 3; **Table 4**). In line with numerous previous reports, single HLA mismatches associated with an increased risk for all endpoints except relapse incidence. Non-permissive HLA-DPB1 mismatches correlated with inferior GRFS and higher aGvHD risk but had no statistically significant impact on either OS or DFS. Last, increasing donor age was also highly predictive of mortality. Other clinically relevant covariates that were included in the final models are reported in **Tables 3**, **4**.

**Table 4:** Competing risk endpoints 10/10 HLA-matched group.

	NRM		aGvHD II-IV		aGvHD III-IV		cGvHD		Relapse	
	HR (95% CI)	p-value	HR (95% CI)	p-value	HR (95% CI)	p-value	HR (95% CI)	p-value	HR (95% CI)	p-value
**Patient age**	1.02 (1.01–1.02)	**<0.001**	1.00 (0.99–1.00)	0.154	1.00 (0.99–1.01)	0.394	–		1.01 (1.00–1.01)	**0.006**
**Donor age 18–30**	1.00		1.00		1.00		–		1.00	
**Donor age 31–45**	1.22 (1.03–1.45)	**0.024**	1.15 (0.96–1.38)	0.126	1.62 (1.20–2.19)	**0.001**	–		1.01 (0.89–1.16)	0.830
**Donor age 46–60**	1.44 (1.15–1.81)	**0.002**	1.28 (1.01–1.62)	**0.040**	1.78 (1.22–2.59)	**0.003**	–		0.85 (0.70–1.02)	0.084
**Early-stage disease**	1.00		–		–		–		1.00	
**Intermediate-stage disease**	1.00 (0.81–1.23)	0.992	–		–		–		1.52 (1.30–1.79)	**<0.001**
**Advanced-stage disease**	1.39 (1.15–1.68)	**<0.001**	–		–		–		1.85 (1.57–2.18)	**<0.001**
***HLA-DRB3/4/5* M, n = 2,075**	1.00		1.00		1.00		1.00		1.00	
***HLA-DRB3/4/5* ARD MM, n = 201**	1.35 (1.05–1.73)	**0.017**	1.16 (0.89–1.52)	0.277	1.19 (0.79–1.79)	0.412	1.12 (0.85–1.47)	0.433	0.84 (0.67–1.06)	0.140
**HLA-DRB4 E3 M, n = 2,268**	1.00		1.00		1.00		1.00		1.00	
**HLA-DRB4 E3 MM, n = 103**	0.95 (0.65–1.39)	0.798	1.26 (0.89–1.78)	0.190	0.95 (0.52–1.74)	0.879	0.92 (0.61–1.37)	0.672	1.10 (0.79–1.53)	0.579
**HLA-DPB1 Match/Permissive**	1.00		1.00		1.00		–		–	
**HLA-DPB1 Non-Permissive**	1.16 (1.00–1.35)	**0.049**	1.32 (1.13–1.55)	**<0.001**	1.40 (1.10–1.80)	**0.007**	–		–	
***In vivo* TCD**	–		1.00		1.00		1.00		1.00	
**No *in vivo* TCD**	–		1.52 (1.26–1.83)	**<0.001**	1.62 (1.22–2.15)	**0.001**	1.80 (1.52–2.14)	**<0.001**	1.24 (1.06–1.45)	**0.007**
**Year of Tx 2000–2003**	1.00		1.00		1.00		–		1.00	
**Year of Tx 2004–2009**	0.46 (0.29–0.72)	**0.001**	0.52 (0.28–0.95)	**0.035**	0.41 (0.17–0.98)	**0.045**	–		1.69 (0.93–3.07)	0.083
**Year of Tx 2010–2014**	0.42 (0.27–0.67)	**<0.001**	0.52 (0.28–0.97)	**0.039**	0.40 (0.16–0.99)	**0.048**	–		1.77 (0.96–3.25)	0.066
**RIC**	1.00		–		–		–		–	
**MAC**	1.27 (1.07–1.50)	**0.006**	–		–		–		–	
**KPS 80–100**	–		–		–		–		1.00	
**KPS <80**	–		–		–		–		1.40 (1.06–1.87)	**0.020**

NRM, non-relapse survival; aGvHD, acute graft-versus-host disease; cGvHD, chronic graft-versus-host disease; M, match; MM, mismatch; ARD, antigen recognition domain; RIC, reduced-intensity conditioning; TCD, T-cell depletion; MAC, myeloablative conditioning; KPS, Karnofsky Performance Score.

Statistical significance is marked in bold.

## Discussion

Histocompatibility assessment for classical HLA (*HLA-A*, *HLA-B*, *HLA-C*, *HLA-DRB1*, *HLA-DQB1*) alleles is currently the consensus for unrelated donor HSCT in Germany (21–24). Among HLA-class II molecules, the *HLA-DRB1* gene shows the highest diversity and highest cell surface expression (25). However, next to the aforementioned highly expressed and polymorphic loci, there are also low expressed genes that appear to be less diverse. Among these are the *HLA-DRB3/4/5* genes, which are genetically linked to the *HLA-DRB1* gene and form the serologically defined *DR52*, *DR53*, and *DR51* antigens. Because of the strong linkage disequilibrium with the *HLA-DRB1* gene, incompatibilities for *HLA-DRB3/4/5* are only observed in a relatively small fraction of transplant pairs when classical HLA alleles are otherwise matched (2). Therefore, there is currently only limited data on the role of *HLA-DRB3/4/5* incompatibility in HSCT. In this study, we sought to investigate this parameter in a retrospective analysis of 3,410 patients who received their first unrelated allogeneic transplant between 2000 and 2014 in a malignant hematologic disease setting.

As shown in **Figure 1A**, *HLA-DRB3/4/5*-incompatible transplants with a mismatch in the ARD region in an otherwise 10/10 HLA-matched setting had a significantly worse OS when compared to *HLA-DRB3/4/5* ARD-compatible transplants. This could be observed in both univariate and multivariate analyses (**Table 3**). *HLA-DRB1* mismatches have shown high relative risks for overall mortality and GvHD incidences in other independent (21, 24) and non-independent large studies (23). This may be partly explained by the fact that DRB1 mismatches often associate with *HLA-DRB3/4/5* mismatches, which in turn exert an additional effect on top of *HLA-DRB1* disparity. Moreover, lower surface expression of these loci compared to *HLA-DRB1* may account for the apparently reduced immunogenicity of *HLA-DRB3/4/5* mismatches. No significant results were seen in the 9/10 HLA-matched setting as shown in **Figure 1B**. The *post-hoc* power for this subanalysis was 44%, indicating that the case numbers in this subgroup were possibly insufficient to show a putative effect in a statistically significant manner.

An impact of *HLA-DRB3/4/5* mismatch on OS has not yet been described in other studies (11, 12). This might be attributed to the relatively high percentage of older patients included in this study (i.e., 30% >60 years and 54 years median age), given that HLA-associated risk increases with age particularly with regard to transplant-related morbidity (26). In our cohort, the effect on OS was in fact mainly driven by an increased NRM (HR 1.35, p = 0.017; **Table 4**). Although statistical significance was not reached in the aGvHD models including only *HLA-DRB3/4/5* ARD-mismatched cases, the analysis in a model encompassing all types of *HLA-DRB3/4/5* mismatches revealed a statistically significant higher risk of aGvHD II–IV in the complete cohort (HR 1.21, 95% CI 1.02–1.43, p = 0.027). The inability to clearly identify this effect in the *HLA-DRB3/4/5* ARD-mismatched cases alone is more likely due to a lack of statistical power rather than lack of association, considering that previous studies have already shown a significantly higher risk of aGvHD in patients receiving *HLA-DRB3/4/5-*mismatched grafts (10, 11). Having said that, an additive effect of HLA-DRB4 E3 mismatches cannot be excluded (11), despite the common perception that mismatches not affecting the ARD have a low impact on alloreactivity (27). According to a recently published study, HLA mismatches outside the ARD that were newly detected after retrospective ultrahigh-resolution HLA genotyping of 5,140 10/10 HLA-matched transplant pairs did associate with a higher risk of aGvHD and TRM but no inferior survival (28). The fact that*HLA-DRB3/4/5* incompatibility increased less the risk of aGvHD when compared to *HLA-DRB1* or other single HLA incompatibility, as reported in this (i.e., HLA mismatch GvHD II–IV: HR 1.38, p < 0.001; **Supplementary Table S4**) but also in other studies conducted in Europe and the United States (21, 24, 29–31), indicates that the effect of *HLA-DRB3/4/5* mismatch is more subtle and thus more difficult to detect.

Regarding the increased NRM observed, it is of note that death due to infections was more frequent in the *HLA-DRB3/4/5* ARD-mismatched group (10/10 HLA: 38.6% vs. 30.7%), something that warrants further investigation (32).

In multivariate analysis, *HLA-DRB3/4/5* incompatibility was associated with a significantly higher risk of GRFS. Contrary to the other endpoints, GRFS is informative of both disease and transplant-related morbidity, which gives a better understanding about the patients’ quality of life after HSCT. Ducreux etal. (11) also described a lower GRFS in *HLA-DRB3/4/5*-mismatched patients—something that was confirmed in this study. As already mentioned, a higher risk of aGvHD in DRB3/4/5-mismatched cases was also observed in our study when all types of DRB3/4/5 mismatches were grouped and analyzed together in the whole of our cohort (n = 3,410).

Our study differs from previously published studies in several aspects. Fernandez-Vina etal. (12) analyzed mismatches in low expressing loci (LEL) alleles in general, namely, without segregating the impact of HLA-DQ or HLA-DP mismatches, which appear to have different immunobiological properties (33–35). We, on the other hand, analyzed the impact of *HLA-DRB3/4/5* mismatches independently from other LEL.

Furthermore, since matching for HLA-DP epitopes has been shown to play an important role in HSCT, we analyzed the distribution of HLA-DP-matched/permissive and non-permissive transplantations among *HLA-DRB3/4/5*-matched and -mismatched cases, respectively (33–35). As shown in **Table 1**, the distribution was similar between the subgroups analyzed, with mild differences observed only in a relatively small fraction of cases (i.e., 5.5% and 8.3% more cases with DPB1 non-permissive mismatches in the DRB3/4/5-mismatched transplant pairs compared to the DRB3/4/5-matched ones in the 10/10 and 9/10 HLA-matched subgroups, respectively). Moreover, no interaction between HLA-DPB1 and *HLA-DRB3/4/5* mismatches was observed in the multivariate analysis, which justified the independent analysis of *HLA-DRB3/4/5* mismatches on the outcome of HSCT. Previously published studies limited their cohort only to HLA-DPB1-mismatched transplants due to the low numbers of HLA-DPB1-matched cases (11). Because of the independent effect of *HLA-DPB1* and *HLA-DRB3/4/5* mismatches, we chose to include HLA-DPB1-matched transplants and to account for both variables in the multivariate analysis.

Limitations of this study were the heterogeneity of the cohort regarding *in vivo* T-cell depletion and the relatively high proportion of older patients, where the impact of HLA mismatches may be more pronounced (26). Furthermore, although various possible *HLA-DRB3/4/5* disparities were identified (**Table 2**), the cohort size precluded analysis at individual combination levels in order to predict the risk for each one of those separately. This means that, unavoidably, the respective HLA-DRB3, -DRB4, and -DRB5 genes were considered in the analysis as one uniform locus, and thus, no comparison between HLA-DRB3, -DRB4, and -DRB5-mismatched cases was sought, considering as well that in some cases, multiple *HLA-DRB3/4/5* mismatches were observed. Last, another limitation of our study was that HLA differences outside the ARD and incompatibilities in additional genes like HLA-DQA, -DPA, -MICA, -MICB, -E, -F, and –G were not considered. Such an approach could perhaps clarify if the associations observed should be attributed to an independent *HLA-DRB3/4/5* mismatch effect or rather an HLA-haplotype incompatibility impact. Moreover, subanalysis for the vector of mismatches could not be performed due to power considerations.

An aspect that could be investigated in future studies is whether factors influencing the expression levels of *HLA-DRB3/4/5* antigens may also influence the effect of *HLA-DRB3/4/5* incompatibilities on HSCT outcome, as this has been shown to be the case for HLA-DP (36, 37).

## Conclusion

In conclusion, our study suggests that *HLA-DRB3/4/5* incompatibilities in a setting of otherwise 10/10 and possibly also 9/10 HLA-matched uHSCT may increase the risk of adverse outcomes especially in more frail patients who face a higher risk of developing transplant-related complications and should therefore be avoided when possible.

## Data Availability Statement

The datasets presented in this article are not readily available because of data safety and confidentiality regulations denoted in the patient consent forms and the application for ethical approval. Requests to access the datasets should be directed to d.fuerst@blutspende.de.

## Ethics Statement

The studies involving human participants were reviewed and approved by the ethical committee of the University of Ulm. Written informed consent to participate in this study was provided by the participants’ legal guardian/next of kin.

## Author Contributions

CT, DE, HS, JM, and DF are principal investigators. They designed the study, performed data analysis/interpretation, and wrote the article. SaF and CN contributed to the data analysis and writing of the article. CT and DE contributed equally. DF and JM contributed equally. UP, ES, TV, EW-D, GW, NK, NM, HE, KS-E, SeF, JC, MK, MD, BH, SK, and MR contributed patients, reviewed the data, and edited the article. All authors contributed to the article and approved the submitted version.

## Funding

This work was supported by the Wilhelm Sander-Stiftung (Grant No. 2018.092.1).

## Conflict of Interest

The authors declare that the research was conducted in the absence of any commercial or financial relationships that could be construed as a potential conflict of interest.

## Publisher’s Note

All claims expressed in this article are solely those of the authors and do not necessarily represent those of their affiliated organizations, or those of the publisher, the editors and the reviewers. Any product that may be evaluated in this article, or claim that may be made by its manufacturer, is not guaranteed or endorsed by the publisher.
