# Supplementary material for: HLA-DRB3/4/5 Matching Improves Outcome of Unrelated Hematopoietic Stem Cell Transplantation
Source: Front Immunol. 2021 Dec 14;12:771449. doi: 10.3389/fimmu.2021.771449 (PMC8712639; doi:10.3389/fimmu.2021.771449)
Supplement: Supplementary file 1 [file Table_1.docx]

Supplementary Material

| **Supp Table 1: Survival endpoints 9/10 HLA matched group** | | | | | | |
| --- | --- | --- | --- | --- | --- | --- |
|  | **OS** | | **DFS** | | **GRFS** | |
|  | HR (95% CI) | p-Value | HR (95% CI) | p-Value | HR (95% CI) | p-Value |
| **Patient age** | 1.02 (1.01-1.03) | **<0.001** | 1.02 (1.01-1.03) | **<0.001** | 1.01 (1.00-1.02) | **0.002** |
| **Donor age 18-30** | 1.00 |  | 1.00 |  | 1.00 |  |
| **Donor age 31-45** | 1.04 (0.85-1.28) | 0.713 | 1.01 (0.84-1.22) | 0.884 | 1.09 (0.92-1.29) | 0.330 |
| **Donor age 46-60** | 1.28 (1.00-1.63) | **0.048** | 1.22 (0.97-1.53) | 0.085 | 1.14 (0.92-1.40) | 0.226 |
| **Early stage disease** | 1.00 |  | 1.00 |  | 1.00 |  |
| **Intermediate stage disease** | 1.20 (0.97-1.47) | 0.087 | 1.39 (1.13-1.71) | **0.002** | 1.11 (0.92-1.33) | 0.272 |
| **Advanced stage disease** | 1.77 (1.44-2.19) | **<0.001** | 1.87 (1.53-2.30) | **<0.001** | 1.43 (1.19-1.72) | **<0.001** |
| **HLA-DRB3/4/5 M, n=854** | 1.00 |  | 1.00 |  | 1.00 |  |
| **HLA-DRB3/4/5 ARD MM, n=133** | 1.17 (0.92-1.50) | 0.198 | 1.09 (0.87-1.37) | 0.446 | 1.19 (0.97-1.46) | 0.101 |
| **HLA-DRB4 E3 M, n=906** | 1.00 |  | 1.00 |  | 1.00 |  |
| **HLA-DRB4 E3 MM, n=57** | 1.25 (0.87-1.79) | 0.229 | 1.20 (0.86-1.69) | 0.288 | 1.06 (0.77-1.45) | 0.737 |
| **HLA-DPB1 Match/Permissive** | - |  | - |  | 1.00 |  |
| **HLA-DPB1 Non-Permissive** | - |  | - |  | 1.19 (1.03-1.37) | **0.018** |
| **Year of Tx 2000-2003** | - |  | - |  | 1.00 |  |
| **Year of Tx 2004-2009** | - |  | - |  | 0.66 (0.42-1.05) | 0.080 |
| **Year of Tx 2010-2014** | - |  | - |  | 0.68 (0.42-1.12) | 0.129 |
| **In-vivo TCD** | - |  | 1.00 |  | 1.00 |  |
| **No in-vivo TCD** | - |  | 1.22 (0.96-1.54) | 0.097 | 1.33 (1.08-1.64) | **0.008** |
| **KPS 80-100** | 1.00 |  | 1.00 |  | 1.00 |  |
| **KPS <80** | 1.92 (1.38-2.66) | **<0.001** | 1.70 (1.24-2.34) | **0.001** | 1.58 (1.18-2.13) | **0.002** |
| **RIC** | 1.00 |  | 1.00 |  | 1.00 |  |
| **MAC** | 1.30 (1.06-1.60) | **0.013** | 1.26 (1.04-1.54) | **0.020** | 1.24 (1.04-1.49) | **0.017** |
| **P-D CMV neg-neg** | 1.00 |  | - |  | - |  |
| **P-D CMV neg-pos** | 1.05 (0.76-1.44) | 0.774 | - |  | - |  |
| **P-D CMV pos-neg** | 1.22 (0.97-1.53) | 0.094 | - |  | - |  |
| **P-D CMV pos-pos** | 1.13 (0.90-1.43) | 0.289 | - |  | - |  |
| Abbreviations: NRM=non-relapse survival, aGvHD=Acute Graft versus Host disease, cGvHD=Chronic Graft versus Host disease, M=Match, MM=Mismatch, ARD=Antigen recognition domain, RIC=Reduced intensity conditioning, TCD=T-cell depletion, MAC=Myeloablative conditioning, KPS=Karnofsky Performance Score | | | | | | |

| **Supp Table 2: Competing risks endpoints 9/10 HLA matched group** | | | | | | | | | | |
| --- | --- | --- | --- | --- | --- | --- | --- | --- | --- | --- |
|  | **NRM** | | **aGvHD II-IV** | | **aGvHD III-IV** | | **cGvHD** | | **Relapse** | |
|  | HR (95% CI) | p-Value | HR (95% CI) | p-Value | HR (95% CI) | p-Value | HR (95% CI) | p-Value | HR (95% CI) | p-Value |
| **Patient age** | 1.03 (1.02-1.04) | **<0.001** | 0.99 (0.99-1.00) | 0.092 | 1.00 (0.99-1.01) | 0.679 | - |  | 1.01 (1.00-1.01) | 0.088 |
| **Donor age 18-30** | 1.00 |  | - |  | - |  | - |  | - |  |
| **Donor age 31-45** | 1.19 (0.93-1.52) | 0.162 | - |  | - |  | - |  | - |  |
| **Donor age 46-60** | 1.68 (1.27-2.22) | **<0.001** | - |  | - |  | - |  | - |  |
| **Early stage disease** | - |  | - |  | - |  | - |  | 1.00 |  |
| **Intermediate stage disease** | - |  | - |  | - |  | - |  | 1.65 (1.28-2.11) | **<0.001** |
| **Advanced stage disease** | - |  | - |  | - |  | - |  | 2.18 (1.70-2.81) | **<0.001** |
| **HLA-DRB3/4/5 M, n=854** | 1.00 |  | 1.00 |  | 1.00 |  | 1.00 |  | 1.00 |  |
| **HLA-DRB3/4/5 ARD MM, n=133** | 1.00 (0.75-1.33) | 0.995 | 1.08 (0.79-1.46) | 0.640 | 1.19 (0.78-1.82) | 0.430 | 1.06 (0.77-1.46) | 0.719 | 1.17 (0.91-1.51) | 0.219 |
| **HLA-DRB4 E3 M, n=906** | 1.00 |  | 1.00 |  | 1.00 |  | 1.00 |  | 1.00 |  |
| **HLA-DRB4 E3 MM, n=57** | 1.53 (1.02-2.29) | **0.039** | 1.28 (0.83-1.98) | 0.270 | 1.34 (0.73-2.48) | 0.347 | 1.40 (0.88-2.22) | 0.160 | 0.84 (0.53-1.31) | 0.435 |
| **HLA-DPB1 Match/Permissive** | 1.00 |  | 1.00 |  | 1.00 |  | - |  | - |  |
| **HLA-DPB1 Non-Permissive** | 1.28 (1.06-1.56) | **0.012** | 1.34 (1.09-1.65) | **0.006** | 1.31 (0.96-1.78) | 0.09 | - |  | - |  |
| **In-vivo TCD** | - |  | 1.00 |  | 1.00 |  | - |  | 1.00 |  |
| **No in-vivo TCD** | - |  | 1.55 (1.21-2.00) | **0.001** | 1.70 (1.20-2.41) | **0.003** | - |  | 1.60 (1.26-2.03) | **<0.001** |
| **Year of Tx 2000-2003** | 1.00 |  | 1.00 |  | 1.00 |  | 1.00 |  | 1.00 |  |
| **Year of Tx 2004-2009** | 0.59 (0.35-0.99) | **0.047** | 0.63 (0.39-1.00) | 0.051 | 0.66 (0.29-1.49) | 0.317 | 0.55 (0.36-0.83) | **0.004** | 6.33 (1.56-25.73) | **0.010** |
| **Year of Tx 2010-2014** | 0.64 (0.37-1.09) | 0.100 | 0.68 (0.42-1.11) | 0.124 | 0.79 (0.35-1.82) | 0.586 | 0.39 (0.26-0.61) | **<0.001** | 6.71 (1.65-27.24) | **0.008** |
| **RIC** | 1.00 |  | - |  | - |  | 1.00 |  | 1.00 |  |
| **MAC** | 1.33 (1.06-1.66) | **0.012** | - |  | - |  | 0.70 (0.55-0.87) | **0.002** | 1.39 (1.12-1.73) | **0.003** |
| **KPS 80-100** | - |  | - |  | - |  | - |  | 1.00 |  |
| **KPS <80** | - |  | - |  | - |  | - |  | 1.52 (1.09-2.11) | **0.013** |
| Abbreviations: NRM=non-relapse survival, aGvHD=Acute Graft versus Host disease, cGvHD=Chronic Graft versus Host disease, M=Match, MM=Mismatch, RIC=Reduced intensity conditioning, TCD=T-cell depletion, MAC=Myeloablative conditioning, KPS=Karnofsky Performance Score | | | | | | | | | | |

| **Supp Table 3: Survival endpoints in the complete group, DRB3/4/5 ARD+HLA-DRB4 E3 Mismatches combined** | | | | | | |
| --- | --- | --- | --- | --- | --- | --- |
|  | **OS** | | **DFS** | | **GRFS** | |
|  | HR (95% CI) | p-Value | HR (95% CI) | p-Value | HR (95% CI) | p-Value |
| **Patient age** | 1.02 (1.01-1.02) | **<0.001** | 1.02 (1.01-1.02) | **<0.001** | 1.01 (1.00-1.01) | **<0.001** |
| **Donor age 18-30** | 1.00 |  | 1.00 |  | - |  |
| **Donor age 31-45** | 1.13 (1.01-1.27) | **0.035** | 1.07 (0.97-1.18) | 0.198 | - |  |
| **Donor age 46-60** | 1.24 (1.07-1.43) | **0.005** | 1.14 (1.00-1.30) | 0.056 | - |  |
| **Early stage disease** | 1.00 |  | 1.00 |  | 1.00 |  |
| **Intermediate stage disease** | 1.21 (1.07-1.36) | **0.002** | 1.34 (1.19-1.51) | **<0.001** | 1.23 (1.10-1.36) | **<0.001** |
| **Advanced stage disease** | 1.83 (1.62-2.07) | **<0.001** | 1.88 (1.67-2.11) | **<0.001** | 1.52 (1.37-1.69) | **<0.001** |
| **HLA 10/10** | 1.00 |  | 1.00 |  | 1.00 |  |
| **HLA 9/10** | 1.25 (1.12-1.38) | **<0.001** | 1.13 (1.03-1.24) | **0.011** | 1.24 (1.13-1.35) | **<0.001** |
| **HLA-DRB3/4/5 M, n=2,929** | 1.00 |  | 1.00 |  | 1.00 |  |
| **HLA-DRB3/4/5 MM, n=481** | 1.17 (1.02-1.34) | **0.022** | 1.10 (0.97-1.25) | 0.132 | 1.14 (1.02-1.28) | **0.023** |
| **HLA-DPB1 Match/Permissive** | - |  | - |  | 1.00 |  |
| **HLA-DPB1 Non-Permissive** | - |  | - |  | 1.15 (1.06-1.25) | **0.001** |
| **Year of Tx 2000-2003** | - |  | - |  | 1.00 |  |
| **Year of Tx 2004-2009** | - |  | - |  | 0.55 (0.40-0.75) | **<0.001** |
| **Year of Tx 2010-2014** | - |  | - |  | 0.55 (0.39-0.76) | **<0.001** |
| **In-vivo TCD** | 1.00 |  | 1.00 |  | 1.00 |  |
| **No in-vivo TCD** | 1.21 (1.05-1.4) | **0.010** | 1.19 (1.05-1.36) | **0.008** | 1.25 (1.11-1.41) | **<0.001** |
| **KPS 80-100** | 1.00 |  | 1.00 |  | 1.00 |  |
| **KPS <80** | 1.64 (1.33-2.04) | **<0.001** | 1.66 (1.37-2.02) | **<0.001** | 1.46 (1.21-1.75) | **<0.001** |
| **RIC** | 1.00 |  | 1.00 |  | 1.00 |  |
| **MAC** | 1.22 (1.08-1.38) | **0.001** | 1.22 (1.09-1.36) | **<0.001** | 1.11 (1.00-1.23) | **0.046** |
| **P-D CMV neg-neg** | 1.00 |  | - |  | - |  |
| **P-D CMV neg-pos** | 1.13 (0.94-1.37) | 0.188 | - |  | - |  |
| **P-D CMV pos-neg** | 1.16 (1.01-1.33) | **0.032** | - |  | - |  |
| **P-D CMV pos-pos** | 1.09 (0.96-1.24) | 0.174 | - |  | - |  |
| Abbreviations: OS=Overall survival, DFS=Disease-free survival, GRFS=GvHD and relapse-free survival, M=Match, MM=Mismatch, TCD=T-cell depletion, KPS=Karnofsky Performance Score, RIC=Reduced intensity conditioning, MAC=Myeloablative conditioning-D=Patient-Donor | | | | | | |

| **Supp Table 4: Competing risks endpoints in the complete group, DRB3/4/5 ARD+HLA-DRB4 E3 Mismatches combined** | | | | | | | | | | |
| --- | --- | --- | --- | --- | --- | --- | --- | --- | --- | --- |
|  | **NRM** | | **aGvHD II-IV** | | **aGvHD III-IV** | | **cGvHD** | | **Relapse** | |
|  | HR (95% CI) | p-Value | HR (95% CI) | p-Value | HR (95% CI) | p-Value | HR (95% CI) | p-Value | HR (95% CI) | p-Value |
| **Patient age** | 1.02 (1.02-1.03) | **<0.001** | 1.00 (0.99-1.00) | **0.045** | 1.00 (0.99-1.00) | 0.369 | - |  | 1.01 (1.00-1.01) | **0.001** |
| **Donor age 18-30** | 1.00 |  | 1.00 |  | 1.00 |  | - |  | - |  |
| **Donor age 31-45** | 1.21 (1.05-1.39) | **0.008** | 1.13 (0.98-1.31) | 0.104 | 1.36 (1.08-1.72) | **0.008** | - |  | - |  |
| **Donor age 46-60** | 1.52 (1.28-1.81) | **<0.001** | 1.22 (1.01-1.47) | 0.038 | 1.47 (1.10-1.96) | **0.008** | - |  | - |  |
| **Early stage disease** | 1.00 |  | - |  | - |  | 1.00 |  | 1.00 |  |
| **Intermediate stage disease** | 1.03 (0.88-1.21) | 0.695 | - |  | - |  | 0.95 (0.80-1.12) | 0.528 | 1.58 (1.38-1.81) | **<0.001** |
| **Advanced stage disease** | 1.32 (1.14-1.54) | **<0.001** | - |  | - |  | 0.85 (0.71-1.01) | 0.073 | 1.95 (1.70-2.24) | **<0.001** |
| **HLA 10/10** | 1.00 |  | 1.00 |  | 1.00 |  | 1.00 |  | - |  |
| **HLA 9/10** | 1.27 (1.12-1.44) | **<0.001** | 1.38 (1.21-1.57) | **<0.001** | 1.58 (1.30-1.93) | **<0.001** | 1.23 (1.07-1.4) | **0.003** | - |  |
| **HLA-DRB3/4/5 M, n=2,929** | 1.00 |  | 1.00 |  | 1.00 |  | 1.00 |  | 1.00 |  |
| **HLA-DRB3/4/5 MM, n=481** | 1.17 (0.99-1.37) | 0.063 | 1.21 (1.02-1.43) | **0.027** | 1.20 (0.93-1.55) | 0.162 | 1.07 (0.90-1.28) | 0.429 | 0.98 (0.84-1.13) | 0.755 |
| **HLA-DPB1 Match/Permissive** | 1.00 |  | 1.00 |  | 1.00 |  | - |  | 1.00 |  |
| **HLA-DPB1 Non-Permissive** | 1.21 (1.08-1.37) | **0.001** | 1.32 (1.17-1.50) | **<0.001** | 1.36 (1.12-1.65) | **0.002** | - |  | 0.91 (0.83-1.01) | 0.079 |
| **In-vivo TCD** | - |  | 1.00 |  | 1.00 |  | 1.00 |  | 1.00 |  |
| **No in-vivo TCD** | - |  | 1.58 (1.37-1.83) | **<0.001** | 1.67 (1.34-2.07) | **<0.001** | 1.53 (1.31-1.78) | **<0.001** | 1.36 (1.19-1.55) | **<0.001** |
| **Year of Tx 2000-2003** | 1.00 |  | 1.00 |  | 1.00 |  | 1.00 |  | 1.00 |  |
| **Year of Tx 2004-2009** | 0.53 (0.37-0.74) | **<0.001** | 0.62 (0.42-0.90) | **0.003** | 0.57 (0.31-1.04) | 0.069 | 0.74 (0.51-1.06) | 0.101 | 2.51 (1.42-4.44) | **0.001** |
| **Year of Tx 2010-2014** | 0.51 (0.36-0.73) | **<0.001** | 0.65 (0.44-0.97) | **0.006** | 0.62 (0.33-1.15) | 0.126 | 0.65 (0.44-0.94) | **0.022** | 2.67 (1.50-4.74) | **0.001** |
| **RIC** | 1.00 |  | - |  | - |  | 1.00 |  | 1.00 |  |
| **MAC** | 1.26 (1.10-1.44) | **0.001** | - |  | - |  | 0.86 (0.75-0.98) | **0.028** | 1.20 (1.07-1.34) | **0.002** |
| **KPS 80-100** | - |  | - |  | - |  | - |  | 1.00 |  |
| **KPS <80** | - |  | - |  | - |  | - |  | 1.47 (1.19-1.83) | **<0.001** |
| Abbreviations: NRM=non-relapse survival, aGvHD=Acute Graft versus Host disease, cGvHD=Chronic Graft versus Host disease, M=Match, MM=Mismatch, RIC=Reduced intensity conditioning, TCD=T-cell depletion, MAC=Myeloablative conditioning, KPS=Karnofsky Performance Score | | | | | | | | | | |

| **Supp Table 5: Examples of DRB3/4/5 Mismatch combinations** | | | |
| --- | --- | --- | --- |
| **DRB345 Status** | **Patient** | **Donor** | **Comment** |
| **3ARD** | DRB3*01:01:02G | DRB3*02:02:01G |  |
| **3ARD, 3ARD** | DRB3*03:01:01G | DRB3*01:01:02G, 02:02:01G | DRB1 matched |
| **3ARD, 4ARD** | DRB3*02:02:01G, DRB4*01:03:01:02N | DRB3*01:01:02G, DRB4*01:03:01:01 | DRB1 matched |
| **3ARD, 4E3** | DRB3*01:01:02G, DRB4*01:01:01:01 | DRB3*02:02:01G, DRB4*01:03:01:01 | DRB1 matched |
| **3ARD, 4E3, 5ARD** | DRB3*02:02:01G, DRB4*01:03:01:01 | DRB4*01:01:01:01, DRB5*01:01:01G | DRB1 Antigen Mismatch |
| **3ARD, 5ARD** | DRB3*02:02:01G, DRB5*01:01:01G | DRB3*01:01:02G | Atypical DRB1*01-DRB5* Association |
| **4ARD** | DRB4*01:01:01:01 | DRB4*01:03:01:02N |  |
| **4ARD, 4ARD** | DRB4*01:03:01:02N | DRB4*01:01:01:01, DRB4*01:03:01:01 | DRB1 matched |
| **4E3** | DRB4*01:01:01:01 | DRB4*01:03:01:01 |  |
| **5ARD** | DRB3*01:01:02G, DRB5*01:01:01G | DRB3*01:01:02G | Atypical DRB1*01-DRB5* Association |
| Abbreviations: The leading number denotes the gene DRB3, DRB4 or DRB5, ARD is a mismatch within the antigen recognition domain, E3 is a mismatch within exon 3. Concomitant mismatches are separated by comma. Shown is the most frequent HLA-mismatch combination within each category | | | |
